# Using CollaboRATE, a brief patient‐reported measure of shared decision making: Results from three clinical settings in the United States

**DOI:** 10.1111/hex.12588

**Published:** 2017-07-05

**Authors:** Rachel C. Forcino, Paul J. Barr, A. James O'Malley, Roger Arend, Molly G. Castaldo, Elissa M. Ozanne, Sanja Percac‐Lima, Cheryl D. Stults, Ming Tai‐Seale, Rachel Thompson, Glyn Elwyn

**Affiliations:** ^1^ The Dartmouth Institute for Health Policy & Clinical Practice Lebanon NH USA; ^2^ Dartmouth‐Hitchcock Patient and Family Advisory Council Lebanon NH USA; ^3^ Dartmouth Master of Health Care Delivery Science Program Hanover NH USA; ^4^ Department of Population Health Sciences University of Utah Salt Lake City UT USA; ^5^ Harvard Medical School Boston MA USA; ^6^ Massachusetts General Hospital Chelsea HealthCare Center Chelsea MA USA; ^7^ Palo Alto Medical Foundation Research Institute Palo Alto CA USA

**Keywords:** patient experience measure, shared decision‐making, survey

## Abstract

**Introduction:**

CollaboRATE is a brief patient survey focused on shared decision making. This paper aims to (i) provide insight on facilitators and challenges to implementing a real‐time patient survey and (ii) evaluate CollaboRATE scores and response rates across multiple clinical settings with varied patient populations.

**Method:**

All adult patients at three United States primary care practices were eligible to complete CollaboRATE post‐visit. To inform key learnings, we aggregated all mentions of unanticipated decisions, problems and administration errors from field notes and email communications. Mixed‐effects logistic regression evaluated the impact of site, clinician, patient age and patient gender on the CollaboRATE score.

**Results:**

While CollaboRATE score increased only slightly with increasing patient age (OR 1.018, 95% CI 1.014‐1.021), female patient gender was associated with significantly higher CollaboRATE scores (OR 1.224, 95% CI 1.073‐1.397). Clinician also predicts CollaboRATE score (random effect variance 0.146). Site‐specific factors such as clinical workflow and checkout procedures play a key role in successful in‐clinic implementation and are significantly related to CollaboRATE scores, with Site 3 scoring significantly higher than Site 1 (OR 1.759, 95% CI 1.216 to 2.545) or Site 2 (z=−2.71, 95% CI −1.114 to −0.178).

**Discussion:**

This study demonstrates that CollaboRATE can be used in diverse primary care settings. A clinic's workflow plays a crucial role in implementation. Patient experience measurement risks becoming a burden to both patients and administrators. Episodic use of short measurement tools could reduce this burden.

## 1 INTRODUCTION

An emphasis in United States (US) health‐care policy on improving patient experiences of care has led to increased focus on structural and process measures of health care, including patient satisfaction with health‐care facilities, personnel and service delivery.^1^, ^2^, ^3^, ^4^ Recently, interest has grown in shared decision making, a process where patients are supported to participate in health‐care decisions. Given this interest, CollaboRATE was developed as a process measure of shared decision making (SDM) between patients and clinicians.^5^, ^6^

Existing patient experience measurement typically involves lengthy surveys that ask patients to recall encounters occurring up to 6 months in the past.^7^ In contrast, CollaboRATE is brief and focuses on a core construct, namely the perception of being informed and then involved in decision‐making steps. This focus reduces survey burden and enables the use of efficient and inexpensive survey administration methods which minimize the delay between a patient experiencing a health‐care interaction and responding to an evaluation survey (eg,. using text messages or automated telephone calls). This real‐time survey administration allows patients to more easily recall their satisfaction with care, and Stull et al.^8^ find recall of satisfaction to be optimal within days of a clinic visit.

Few studies measure outpatient care experiences close to the time of visit and those that do are often conducted in the context of a specific disease.^9^, ^10^ One exception is found in Tai‐Seale's^11^ cluster randomized pilot trial of recently developed SDM interventions, where SDM was assessed immediately post‐visit. Further, adding a quality improvement perspective to data collection requirements poses additional feasibility challenges. Instead of deploying dedicated research staff to ensure survey completion, the measurement of patient experience in usual practice settings may require clinic staff to take on new tasks in addition to their existing workloads. Despite widespread patient survey administration as part of clinical operations, the available literature provides little insight on how to best administer real‐time patient surveys. Emerging research from Carter et al.^12^ begins to address this issue in the United Kingdom's National Health Service; the current paper expands on this effort by incorporating insights from diverse US practices.

In light of the emphasis placed on process measures of patient experience and the paucity of reports addressing feasibility and results of routine, real‐time, patient‐reported measurement of shared decision making across multiple sites, we aim to (i) provide insight on facilitators and challenges to implementing a real‐time patient survey and (ii) evaluate CollaboRATE scores and response rates across multiple primary care clinical settings with varied patient populations.

## 2 METHOD

As part of a quality improvement initiative, we collected CollaboRATE post‐visit survey data at three geographically and demographically varied primary care practices within the United States: Lebanon in New Hampshire, Los Gatos in California and Chelsea in Massachusetts.

### 2.1 Data

#### 2.1.1 CollaboRATE

The CollaboRATE questions are as follows: (i) How much effort was made to help you understand your health issues? (ii) How much effort was made to listen to what matters most to you about your health issues? (iii) How much effort was made to include what matters most to you in choosing what to do next? CollaboRATE survey data included responses to the three CollaboRATE questions, each on a 0‐9 scale, along with each respondent's age, gender and clinician. CollaboRATE has previously been validated in a simulation sample^5^ and included significant end‐user input in its development.^6^

#### 2.1.2 Field notes

Field notes regarding implementation challenges and solutions were kept on an ad hoc basis by study staff at all sites throughout the project as an integral part of project management processes. Investigators and research staff from all sites contributed observations through email messages and regular project meetings; meetings were held weekly at Site 1 and quarterly between Sites 1 and 2 and Sites 1 and 3. All contributors were familiar with the study protocol and participated in the design of survey implementation at their respective sites. Field notes were collected, organized and held by the project coordinator based at the Lebanon, NH site. All instances of unanticipated decisions, problems and errors were documented.

### 2.2 Settings, participants and data collection processes

#### 2.2.1 Overview

##### Setting

Three geographically diverse US primary care practices were included in this study: Lebanon, NH (Site 1); Los Gatos, CA (Site 2); and Chelsea, MA (Site 3). Detailed information on each site is included below.

##### Participants

All adult patients ages 18 and older visiting the participating primary care teams were eligible to participate.

##### Data collection process

All sites allowed clinicians to describe the survey to patients, although this behaviour was eventually encouraged as a participation‐enhancing measure only at the Los Gatos site. No clinicians at any site personally delivered the survey to their patients.

#### 2.2.2 Site 1: Lebanon, New Hampshire

##### Setting details

This setting included three primary care teams based in a rural academic medical centre in New Hampshire with approximately 16 000 patients. Clinicians included physicians, physicians’ assistants, nurses, nurse practitioners and pharmacists. The patient population is more than 95% white and non‐Hispanic, and 99% of patients speak English as a primary language.

##### Participant details

Parents and guardians of patients under 18 were also eligible to complete CollaboRATE on behalf of their children at this site.

##### Data collection process

This site aimed to administer CollaboRATE for 15 consecutive months beginning in April 2014 in order to embed the survey in routine practice. Administrative staff were asked to notify patients at check‐in of the ongoing research study. The study was reviewed by the Dartmouth Committee for the Protection of Human Subjects and written participant consent requirements were waived for all modes of survey administration. Five survey administration modes were implemented consecutively: (i) paper survey in‐clinic from April through July 2014; (ii) patients were alerted to a survey hosted on the online patient portal (MyChart) of the Epic electronic medical record system from July through November 2014; (iii) automated interactive voice response (IVR) telephone calls were made to patients’ cellular telephones from December 2014 through March 2015; (iv) short messaging service (SMS) text messages were sent to patients’ cellular telephones from April through July 2015; (v) a tablet computer survey was presented to patients in‐clinic, with an option for patients to mail back a paper‐based survey if they declined to complete the tablet survey (July through October 2015); see Appendix 1 for detail. The study population at the Lebanon, NH site included all patients for whom institutional constraints did not explicitly bar survey delivery, that is all patients during paper delivery mode, all patients with online patient portal accounts in the MyChart mode, and patients with cellular telephone numbers on record in the IVR and SMS modes.

#### 2.2.3 Site 2: Los Gatos, California

##### Setting details

This primary care clinic in suburban Los Gatos, CA is one of many community‐based clinics of the Palo Alto Medical Foundation, a large, not‐for‐profit health‐care delivery system in the San Francisco Bay Area, and has a patient panel of approximately 13 000. The patient population is predominantly Caucasian (44%) and Asian (34%). Approximately 90% of patients speak English as a primary language, with 46 other language groups also represented.

##### Data collection process

This site aimed to collect 300 completed CollaboRATE surveys during the February 2015‐March 2015 study period. The study was reviewed by the Sutter Health Institutional Review Board and written participant consent requirements were waived. An initial week‐long pilot period involved only the reception staff informing patients about the CollaboRATE survey as they arrived for their visit. After their visit, a receptionist invited all eligible patients to complete a paper survey containing CollaboRATE. The pilot period yielded very low response rates (2%). Recruitment in the subsequent data collection period included asking physicians to encourage participation, additional reminder signage, and medical assistants providing patients with surveys before they left the exam room. A locked collection box was placed at the clinic exit with a sign reminding patients to deposit the completed survey in the box. Thus, the physical clinic layout did not present a barrier to survey completion at this site. The study population at the Los Gatos, CA site included all patients visiting the participating clinics.

#### 2.2.4 Site 3: Chelsea, Massachusetts

##### Setting details

This urban adult medicine practice is based in a Massachusetts General Hospital affiliated community health centre in Chelsea, MA and has a patient panel of approximately 14 000 patients. Fifty percent of patients are Latino, 31% white, 9% black and 4% other. Fifty‐one percent of patients at this site speak English as their primary language, while 38% speak only Spanish and 11% speak one of 14 other languages.

##### Data collection process

This site aimed to administer the CollaboRATE survey for the month of May 2015. The study was reviewed by the Partners Institutional Review Board and written participant consent requirements were waived. Medical assistants (MAs) assigned to meet with patients following their visits delivered the survey to patients. Survey forms were available in both English and Spanish.^13^ Completed surveys were placed in a secure box at the clinic's exit. To encourage uptake, the Chelsea clinic provided each participating MA with a $100 one‐time payment. The study population at the Chelsea, MA site included all patients visiting the participating clinic.

### 2.3 Analysis

Returned surveys missing one or more CollaboRATE responses were considered incomplete and excluded from analysis. Descriptive statistics compared response rates and CollaboRATE scores across sites. Because patient reported experience measures often show ceiling effects, we decided a priori to conduct a top score analysis which has been shown to enhance variation in scores^5^^,.^ 14 Therefore, the CollaboRATE score represents the proportion of patients (minimum sample size of 25) responding with the highest possible score on all three questions. The unit of analysis was patients.

As data were clustered by site and clinician, we used mixed‐effects logistic regression analysis to evaluate the impact of site and clinician on the dichotomous CollaboRATE score outcome variable (ie, top score for all three questions or not top score) while controlling for the survey administration modality and patient‐level demographic characteristics of age and gender. Clinician was included as a random effect within the mixed‐effects regression model (with resulting clinician variance estimate) to account for clustering of responses and to allow our results to be generalizable to the population of all clinicians who would plausibly work at one of the three sites, not just those in our study, as the random effect specification accounts for the possibility that studying a different group of clinicians may have yielded different results. Site was included as a fixed effect because the small number of sites (3) provided insufficient numbers of sites to reliably estimate a site‐level variance component under a random‐effects specification. Thus, we control for the sites in the study rather than generalizing results to a larger population of sites. While the logistic regression model allows us to compare Los Gatos and Chelsea sites to the Lebanon reference group, the logistic regression model does not allow us to test the difference between scores at the Los Gatos and Chelsea sites. Therefore, as a post‐estimation hypothesis test, we calculated a z‐score to compare CollaboRATE scores at the Los Gatos and Chelsea sites. An inverse logit transformation involving the clinician random effect variance parameter allowed the magnitude of the clinician effect to be compared to that of the model's regression parameters on the probability scale.^15^ To assess effect modification by site, we reran the original mixed‐effects logistic regression model described above, this time including two interaction terms accounting for associations between site and patient age or gender, respectively. Survey data was analysed with Stata 13 software.^16^

To inform the key learnings and descriptions of site‐level characteristics, we aggregated all mentions of unanticipated decisions, problems, and administration errors from field notes and email communications.

## 3 RESULTS

### 3.1 Response rates by site

Response rates varied across sites, with Site 3 (Chelsea, MA) achieving the highest response rate of 73% compared to 25% overall at Site 1 (Lebanon, NH) and 30% at Site 2 (Los Gatos, CA). Site 1 saw variation in response rates across the various administration modes, described in detail elsewhere.^17^ Of all surveys returned, missing CollaboRATE data were minimal: <0.5% at Site 1, <1% at Site 2, and <0.1% at Site 3.

### 3.2 Factors influencing CollaboRATE scores

Table 1 demonstrates variation in CollaboRATE score by site, with Site 1 (Lebanon, NH) achieving an overall score of 68% and response rate of 25%, Site 2 (Los Gatos, CA) achieving an overall score of 76% and response rate of 30%, and Site 3 (Chelsea, MA) achieving an overall score of 86% and response rate of 73%.

**Table 1:** Site‐level CollaboRATE scores and response rates

	Overall score(%)	Clinician score range(%)	Response rate(%)	Sample size	Population size	Participating clinicians (n)
Site 1: Lebanon, NH	68	42 ‐ 93	25	4421	17568	34
Mode 1	81	72 ‐ 93	12	541	4692	–
Mode 2	71	59 ‐ 83	34	1019	3015	–
Mode 3	61	42 ‐ 75	25	893	3589	–
Mode 4	65	46 ‐ 82	23	757	3329	–
Mode 5	66	53 ‐ 83	41	1211	2943	–
Site 2: Los Gatos, CA	76	66 ‐ 91	30	323	1094	12
Site 3: Chelsea, MA	86	76 ‐ 99	73	1230	1687	18

While Los Gatos patients were similar to Lebanon patients in their propensity to give a top score on all three CollaboRATE items, Chelsea patients were more likely than those at the Lebanon (OR 1.759, 95% CI 1.216 to 2.545) and Los Gatos (z=−2.71, 95% CI −1.114 to −0.178, *P*=.007) sites to give all top scores. The clinician random effect variance of 0.146 implies that the distribution of CollaboRATE scores varied substantially between clinicians; for this study the random effect standard deviation of 0.382 translates to a difference of 0.5364 on the probability scale. Thus, a clinician whose scores fall one standard deviation above the mean clinician will have a 53.64% greater probability of obtaining a perfect CollaboRATE score from a randomly selected patient.

Patient demographics also play a role. Table 2 shows that while CollaboRATE score increased only slightly with increasing patient age (OR 1.018, 95% CI 1.014 to 1.021), female patient gender was associated with significantly higher CollaboRATE scores across the three sites (OR 1.224, 95% CI 1.073 to 1.397). Our effect modification analysis including interaction terms for associations between site and gender and age variables yielded no significant interactions, suggesting the effect of patient age and gender on CollaboRATE scores is not substantially moderated by site (see Appendix 2).

**Table 2:** Characteristics contributing to variation in CollaboRATE scores: mixed‐effects logistic regression

Random effects	Variance estimate	95% Confidence interval	
Clinician	0.146	0.076	0.282

Fixed effects^a^	Odds ratio	95% Confidence interval	
Site 1: Lebanon, NH (reference)	1.000	–	–
Site 2: Los Gatos, CA	0.922	0.570	1.492
Site 3: Chelsea, MA	1.759	1.216	2.545
Patient age	1.018	1.014	1.021
Patient gender: Female	1.224	1.073	1.397
Constant	1.452	1.036	2.034

a Odds ratios for survey administration modes are available upon request.

### 3.3 Key learnings and site‐level characteristics

The following key learnings and other site‐level characteristics may contribute to the observed variation in CollaboRATE response rates and CollaboRATE scores by site.

#### 3.3.1 Site 1: Lebanon, New Hampshire

The relatively novel patient survey administration procedures used in patient portal, IVR, and SMS modes presented logistical challenges. We relied on the medical centre's information systems department for programming key aspects of survey administration. We were not able to negotiate priority status for our programming needs, given other competing deadlines in the organizational work schedule, and this led to delays and errors. For example, limitations of existing software meant survey format was not exactly as we had stipulated and we were unable to ensure that the invitation to complete the survey on the patient portal was sent from a neutral source and not from their own clinician. We concluded that unless collecting patient experience data was an organizational priority, other organizations’ information systems may be reluctant to facilitate in‐house digital methods of patient survey administration. Capacity for engaging external contractors or an on‐staff programmer might have eliminated these delays, although the need for integration into existing administrative systems would still exist.

In the paper survey administration mode, patients who did not need to schedule follow‐up appointments often did not make the effort to collect the CollaboRATE survey from the assigned staff person, despite this process being intended to occur for each patient. The location of the staff assigned to distribute surveys was not convenient, as their offices were not located near the clinic exit and access by patients often required exiting the consultation room and walking away from the clinic exit. As such, many patients may not have received or completed CollaboRATE due to the clinic layout.

Our attempts to use text messaging (SMS) on cellular telephones also revealed logistical challenges due to significant variation in subscriber plans and very limited reception in some rural areas. In the United States, some cellular telephone subscribers pay a fee (<$0.50 USD) for each text message sent or received. Some cellular service providers offer to deliver an organization's outgoing messages to their customers free of charge for an annual $25 000 USD up‐front fee, although that cost was prohibitive in this project. Despite the cost to patients, we used the SMS approach to assess text message patient survey administration in this study.

#### 3.3.2 Site 2: Los Gatos, California

At the Los Gatos site, the clinic's physicians and operational leaders strongly supported the project. When the initial attempt to collect responses by asking receptionists to alert patients to the survey led to only 26 surveys completed over a two‐week pilot period, we were able to consult with the two clinic leaders and change the survey administration workflow. The new workflow ensured that both clinicians and reception staff were requesting survey completion, supported by the medical assistants giving surveys to patients as they finished their clinic visits. Using the modified recruitment methods, 323 surveys were completed during a two‐week data collection period.

#### 3.3.3 Site 3: Chelsea, Massachusetts

We observed significant staff commitment to the data collection process at this site. The clinic workflow and layout facilitated data collection; patients were required to make contact with administrative staff as they left the clinic, which provided an opportunity for staff to confirm receipt of the survey. We also believe that the additional financial incentive to MAs contributed to the 73% response rate achieved in this site.

## 4 DISCUSSION

### 4.1 Principal findings

Variation was found across the three sites with regard to both response rates and CollaboRATE scores. Site‐level factors were associated with scores at Site 3 (Chelsea) where response rates were highest, but these factors were not as influential as the clinician seen and the patient's gender in accounting for observed variation in CollaboRATE scores. These generic site‐level and clinician‐level variables would include, as a component, the site or clinician's actual level of performance. The range of scores at the clinician level indicates that the measure discriminates between high and low performing clinicians. Additionally, associations between CollaboRATE scores and patient age and gender, respectively, show that older patients are only slightly more likely to give higher CollaboRATE scores than their younger peers, while women are much more likely than men to do so.

Our qualitative assessment found that site‐level factors such as patient flows, physical clinic layout, and staff enthusiasm towards the project led to different response rates. Efforts to collect patient experience data close to clinical encounters need to negotiate two key challenges. First, scores are observed to have a wider range when CollaboRATE is completed outside the clinic environment. This may be due to social desirability bias where patients perceive in‐clinic survey completion to be less private than completion elsewhere. Recency of the visit may also play a role in score differences observed when CollaboRATE is completed inside the clinic immediately after an appointment versus outside the clinic a short time (less than 24 hours) later. Second, efficient interfaces are needed between modern methods of collecting data using online and mobile technologies and the administrative systems of health‐care organizations. Existing systems are not currently designed to allow efficient communication with groups of patients. Adopting methods to assess, analyse and use patient experience data as inputs into quality improvement at the clinician and clinic level will always be difficult unless real‐time survey administration solutions requiring fewer in‐clinic human resources are developed.

### 4.2 Strengths and limitations

The participating sites represent a diverse group of rural, urban, and suburban primary care clinics in which CollaboRATE was successfully administered immediately following primary care clinic visits using multiple survey distribution modalities. Few studies have considered the challenges of collecting real‐time patient experience data in such depth. Our data collection processes sought responses from all adult patients in real‐world primary care practice, potentially avoiding selection bias that may result from collecting survey responses only from those patients who formally document written consent to participate in a research study; however, our lack of demographic data on non‐respondents precludes definitive conclusions about selection bias. Additionally, the short length of the survey reduces the time burden placed on patients as a result of routine data collection.

Our lack of detailed patient demographic data may inflate estimates of site and clinician impact on CollaboRATE scores.^18^, ^19^, ^20^, ^21^ The variation in CollaboRATE score we observed between sites may therefore be due in part to the socio‐demographic diversity of the respondent populations between sites or to selection bias due to differences in response rates between sites, rather than to other potential site‐specific sources of variation such as clinic layout and workflow. Additionally, we lack the ability to link survey responses to individual patients and therefore cannot model survey response through regression analysis. Demographic and performance data on clinicians, if it were available, may also help explain the existing relationship between clinician identity and CollaboRATE scores. Finally, we lack data on those patients at Los Gatos who were unable to participate because the survey was not available in their preferred language, although field notes did not include record of patients unable to complete the survey due to language restrictions.

### 4.3 Context within existing literature

This work contributes to a nascent area of inquiry surrounding real‐time measurement of patient experience, particularly related to shared decision making. Tai‐Seale's^11^ overall CollaboRATE score of 72% is similar to the scores found in the current study, although that work focuses more on assessment of shared decision‐making interventions than on our current question concerning feasibility of real‐time measurement implementation for quality improvement purposes. We found further support for Carter's^12^ work highlighting the challenge of collecting real‐time patient‐reported data following a clinical visit, where patients often face time constraints that lead them not to complete real‐time patient experience measures. These constraints highlight the difficulty of collecting post‐visit surveys in clinic, as compared to screening measures commonly collected in clinic waiting rooms prior to clinic visits such as the PHQ‐2 for depression. SDM interventions, such as decision aids, also face barriers to implementation due in part to uncertainty about the effect of decision aid use on length of the clinical consultation.^22^

### 4.4 Implications

#### 4.4.1 Practice implications

This study demonstrates that a clinic's workflow, especially its patient checkout procedure, plays a key role in successful in‐clinic implementation of a patient experience measure. Ensuring staff commitment to survey administration is a key issue in maximizing response rates to in‐clinic patient surveys. Strong clinical leadership and engagement may play a role in enhancing staff commitment to survey administration, as may financial incentives to staff members required to go above and beyond their usual duties. For technological solutions, efficient and seamless integration with administrative systems is a key requirement. Avoiding the burden of long‐term data collection (as in Site 1) in favour of sampling for shorter time periods (as in Sites 2 and 3) seems to lead to better response rates. Technological survey administration also helps boost response rates.

#### 4.4.2 Policy implications

There is a significant risk that patient experience measurement could become a burden to both patients and administrators unless efforts are made to make the process efficient, time‐limited, and most of all, relevant. Existing outpatient surveys, such as the Clinician and Group Consumer Assessment of Healthcare Providers & Systems (CG‐CAHPS),^7^ measure important elements of patient‐centred care including satisfaction with communication, facilities and clinic staff. However, CG‐CAHPS lacks items directly assessing the process of SDM and takes the form of a lengthy questionnaire.^7^ This level of measurement cannot be sustained unless there is interest in using the data for quality improvement, and it is helpful for the motivation for such use to arise from the organization undertaking the measurement. Episodic use of short measurement tools could reduce this burden.

We conclude that collecting real‐time data about key aspects of patient experience is not easy. Ensuring that the data are made available for clinicians and management so that it can be used for timely quality improvement is a research frontier yet to be explored.

## CONFLICTS OF INTEREST

Financial: Glyn Elwyn reports personal fees from Emmi Solutions LLC, National Quality Forum, Washington State Health Department, Oxford University Press and Radcliffe Press. Non‐Financial: Glyn Elwyn initiated the Option Grid^™^ patient decision aids collaborative, which produces and publishes patient knowledge tools; he has been a member of teams that have developed measures of shared decision making and care integration.

## Appendix 1 Detailed descriptions of survey administration modes

### 1.1

**Table:**

Mode 1: Paper in‐clinic survey	After their visits, patients visited a member of the clinic's administrative staff to receive after‐visit summaries and to schedule potential follow‐up visits. They were given the CollaboRATE survey at this point by the administrative staff person and asked to leave completed surveys in locked survey receptacles.
Mode 2: Patient portal online survey	We delivered CollaboRATE using an online patient portal (MyChart) survey, part of the clinic's electronic medical record. Programming was performed by the medical centre's information systems department. As clinical encounters were completed, emails containing a web link to the CollaboRATE survey were automatically sent to patients who had portal accounts.
Mode 3: Interactive voice response (IVR)	CollaboRATE was delivered to patients by telephone using an interactive voice response system, programmed by the medical centre's information systems department. An automated telephone call was made to each patient's cell phone number at 7:00 pm on the day of their clinic visit. Before initiating the survey, the respondent was asked to confirm that he or she was the individual who had visited the clinic that day. Upon confirmation, numerical keypad responses to CollaboRATE questions were requested. If any of the three CollaboRATE questions remained incomplete at 7:00 pm the following day, an identical automatic call was placed at that time.
Mode 4: Short message service (SMS text messages)	Text messages were sent to patient cell phones at 7:00 pm on the day of their clinical visits, programmed by the medical centre's information systems department. The first message introduced the survey and offered an opt out opportunity. Remaining messages each contained a single CollaboRATE question and response instructions. Subsequent messages were triggered by each further reply. If any of the three CollaboRATE questions remained incomplete at 7:00 pm the following day, the first introductory text message was re‐sent.
Mode 5: Tablet and mail	Using tablet computers, research assistants offered patients an opportunity to complete an online version of CollaboRATE as they left the clinic, hosted in Qualtrics (Qualtrics LLC, Provo, UT). Patients who declined the tablet opportunity were asked to complete and return a paper‐based survey by mail in a postage‐paid envelope.

## Appendix 2 Characteristics contributing to variation in CollaboRATE scores: mixed‐effects logistic regression with interactions by site

### 2.1

**Table:**

Random effects	Variance estimate	95% Confidence interval	
Clinician	0.143	0.074	0.276

Fixed effects^a^	Odds ratio	95% Confidence interval	
Site 1: Lebanon, NH (reference)	1.000	–	–
Site 2: Los Gatos, CA	0.928	0.365	2.360
Site 3: Chelsea, MA	1.576	0.789	3.151
Patient age	1.018	1.014	1.022
Female patient gender	1.181	1.020	1.367
Constant	1.476	1.042	2.090

Interactions	Odds ratio	95% Confidence interval		Χ^2^ b	*P* b
Female patient gender by Site				2.41	.299
Female patient gender x Site 2	1.567	0.880	2.789		
Female patient gender x Site 3	1.093	0.737	1.621		
Patient age by Site				0.48	.785
Patient age x Site 2	0.995	0.979	1.011		
Patient age x Site 3	1.001	0.990	1.012		

a Odds ratios for survey administration modes are available upon request.

b two degree‐of‐freedom tests of whether there was a difference from odds ratio of 1 for the two interaction contrasts whose effects are identified (Sites 2 and 3) against the baseline interaction contrast (of Site 1).

## References

[1] 111th United States Congress . The Patient Protection and Affordable Care Act.; 2010.

[2] Institute of Medicine (US) Committee on Quality of Health Care in America . Crossing the Quality Chasm. Washington: National Academies Press (US); 2001 https://doi.org/10.17226/10027.

[3] Donabedian A . The quality of care: How can it be assessed? JAMA. 1988;260:1743–1748. https://doi.org/10.1001/jama.1988.03410120089033. 3045356 10.1001/jama.260.12.1743

[4] Donabedian A . The quality of medical care. Science. 1978;200:856‐864. https://doi.org/10.1126/science.417400. 417400 10.1126/science.417400

[5] Barr PJ , Thompson R , Walsh T , Grande SW , Ozanne EM , Elwyn G . The psychometric properties of CollaboRATE: a fast and frugal patient‐reported measure of the shared decision‐making process. J Med Internet Res. 2014;16:e2 https://doi.org/10.2196/jmir.3085. 24389354 10.2196/jmir.3085 PMC3906697

[6] Elwyn G , Barr PJ , Grande SW , Thompson R , Walsh T , Ozanne EM . Developing CollaboRATE: a fast and frugal patient‐reported measure of shared decision making in clinical encounters. Patient Educ Couns. 2013;93:102‐107. https://doi.org/10.1016/j.pec.2013.05.009. 23768763 10.1016/j.pec.2013.05.009

[7] Agency for Healthcare Research and Quality . Clinician & Group Survey and Instructions. Available at: http://www.ahrq.gov/cahps/surveys-guidance/cg/instructions/index.html. Accessed September 30, 2016.

[8] Stull DE , Leidy NK , Parasuraman B , Chassany O . Optimal recall periods for patient‐reported outcomes: challenges and potential solutions. Curr Med Res Opin. 2009;25:929‐942. https://doi.org/10.1185/03007990902774765. 19257798 10.1185/03007990902774765

[9] Armes J , Wagland R , Finnegan‐John J , Richardson A , Corner J , Griffiths P . Development and testing of the patient‐reported chemotherapy indicators of symptoms and experience. Cancer Nurs. 2014;37:E52‐E60. https://doi.org/10.1097/NCC.0b013e3182980420. 24141376 10.1097/NCC.0b013e3182980420

[10] Land L , Sizmur S , Harding J , Ross JDC . Development of a validated patient satisfaction survey for HIV clinic attendees. Int J STD AIDS. 2013;24:201‐209. https://doi.org/10.1177/0956462412472447. 23535353 10.1177/0956462412472447

[11] Tai‐Seale M , Elwyn G , Wilson CJ , et al. Enhancing shared decision making through carefully designed interventions that target patient and provider behavior. Health Aff (Millwood). 2016;35:605‐612. https://doi.org/10.1377/hlthaff.2015.1398. 27044959 10.1377/hlthaff.2015.1398

[12] Carter M , Davey A , Wright C , et al. Capturing patient experience: a qualitative study of implementing real‐time feedback in primary care. Br J Gen Pract. 2016;66:e786‐e793. 27621292 10.3399/bjgp16X687085 PMC5072916

[13] Forcino RC , Bustamante N , Elwyn G , Percac‐Lima S , Thompson R , Barr PJ . Translating CollaboRATE: a Spanish version for use in the United States PLoS ONE. 2016;11:e0168538 https://doi.org/10.1371/journal.pone.0168538. 10.1371/journal.pone.0168538 PMC5176178 28002422

[14] Makoul G , Krupat E , Chang C‐H . Measuring patient views of physician communication skills: development and testing of the Communication Assessment Tool. Patient Educ Couns. 2007;67:333‐342. https://doi.org/10.1016/j.pec.2007.05.005. 17574367 10.1016/j.pec.2007.05.005

[15] Austin PC . The performance of different propensity score methods for estimating marginal odds ratios. Stat Med. 2007;26:3078‐3094. https://doi.org/10.1002/sim.2781. 17187347 10.1002/sim.2781

[16] StataCorp . Stata Statistical Software: Release 13. 2013.

[17] Barr PJ , Forcino RC , Thompson R , et al. Evaluating CollaboRATE in a clinical setting: analysis of mode effects on scores, response rates, and costs of data collection. BMJ Open. 2017;7:e014681 https://doi.org/10.1136/bmjopen-2016-014681. 10.1136/bmjopen-2016-014681 PMC5372080 28341691

[18] Xiao H , Barber JP . sx. Value Heal. 2008;11:719‐725. https://doi.org/10.1111/j.1524-4733.2007.00294.x. 10.1111/j.1524-4733.2007.00294.x 18179667

[19] Haviland MG , Morales LS , Dial TH , Pincus HA . Race/ethnicity, socioeconomic status, and satisfaction with health care. Am J Med Qual. 2005;20:195‐203. https://doi.org/10.1177/1062860605275754. 16020676 10.1177/1062860605275754 PMC1781358

[20] Mazor KM , Clauser BE , Field T , Yood RA , Gurwitz JH . A demonstration of the impact of response bias on the results of patient satisfaction surveys. Health Serv Res. 2002;37:1403‐1417. 12479503 10.1111/1475-6773.11194 PMC1464019

[21] Murray‐Garcia JL , Selby JV , Schmittdiel J , Grumbach K , Quesenberry CP Jr . Racial and ethnic differences in a patient survey: patients’ values, ratings, and reports regarding physician primary care performance in a large health maintenance organization. Med Care. 2000;38:300‐310. 10718355 10.1097/00005650-200003000-00007

[22] Stacey D , Légaré F , Col NF , et al. Decision aids for people facing health treatment or screening decisions. Cochrane Database Syst Rev. 2011;CD001431. https://doi.org/10.1002/14651858.CD001431.pub3. 10.1002/14651858.CD001431.pub3 21975733

